# Primary osseous malignancies of the foot: a comprehensive literature review and insights from a single-centre experience

**DOI:** 10.1016/j.jbo.2026.100755

**Published:** 2026-03-12

**Authors:** Juul M.J. Kruijer, Gitte G.J. Krebbekx, Jos A.M. Bramer, Floortje G.M. Verspoor

**Affiliations:** a Department of Orthopaedic Surgery And Sports Medicine, Amsterdam UMC, Amsterdam Movement Sciences, University of Amsterdam, Meibergdreef 9, Amsterdam, the Netherlands; b Cancer Center Amsterdam, Meibergdreef 9, Amsterdam, the Netherlands; c Amsterdam Movement Sciences, Rehabilitation & Development, Meibergdreef 9, Amsterdam, the Netherlands

**Keywords:** Primary malignant bone tumor, Foot, Diagnostic delay, Amputation, Limb salvage

## Abstract

**Aims:**

Primary malignant bone tumors of the foot are rare, with limited and heterogeneous data on.

characteristics, management, and outcomes. This study aims to provide an overview of the epidemiology, treatment, and prognosis of these tumors through a systematic review and cohort analysis.

**Methods:**

PubMed, Embase, and Web of Science were searched for studies on osteosarcoma, Ewing’s sarcoma, and chondrosarcoma of the foot. Studies in English or German including ≥ 10 patients with original data were eligible. Demographic, clinical, treatment, and outcome data were extracted. Additionally, a retrospective review was performed of all patients with foot malignancies (chondrosarcoma, Ewing sarcoma or osteosarcoma) treated at Amsterdam UMC (2013–2024). Data were pooled for analysis.

**Results:**

Eighteen studies met the inclusion criteria, comprising 884 patients including the single center cohort. Osteosarcoma, Ewing’s sarcoma, and chondrosarcoma were the most common malignancies, most frequently affecting the calcaneus, metatarsals, and phalanges, respectively. The median diagnostic delay was 9 (IQR 6–14) months, with pain and swelling as the predominant presenting symptoms. Below-knee amputation was the most common surgical procedure, particularly for hindfoot tumors. Local recurrence rates were highest in osteosarcoma (19%), while Ewing’s sarcoma had the highest incidence of metastatic disease (56%). Disease-specific mortality at last follow-up was 40% for osteosarcoma, 50% for Ewing’s sarcoma, and 21% for chondrosarcoma. The single-centre cohort mirrored these findings, highlighting the challenges of delayed diagnosis and inadvertent surgery.

**Conclusion:**

Primary malignant bone tumors of the foot present significant diagnostic and therapeutic challenges, with high rates of recurrence and metastasis. Early recognition, referral to specialized centers, and multidisciplinary management are essential to improve outcomes. Future research should focus on prospective data collection, standardized reporting, and patient-reported outcomes to guide optimal care.

## Highlights

• Primary malignant bone tumors of the foot are exceptionally rare, leading to frequent diagnostic delays and under-recognition.
• Osteosarcoma and Ewing sarcoma are the most prevalent subtypes, both in the literature and in our single-centre cohort.
• A median diagnostic delay exceeding 6 months was observed, highlighting the need for increased clinical vigilance.
• Amputation remains the most common surgical treatment, although limb-salvage may be possible in selected cases.
• Oncologic outcomes are comparable to tumors in other skeletal sites when timely diagnosis and appropriate treatment are achieved.

## Graphical abstract

**Figure:**
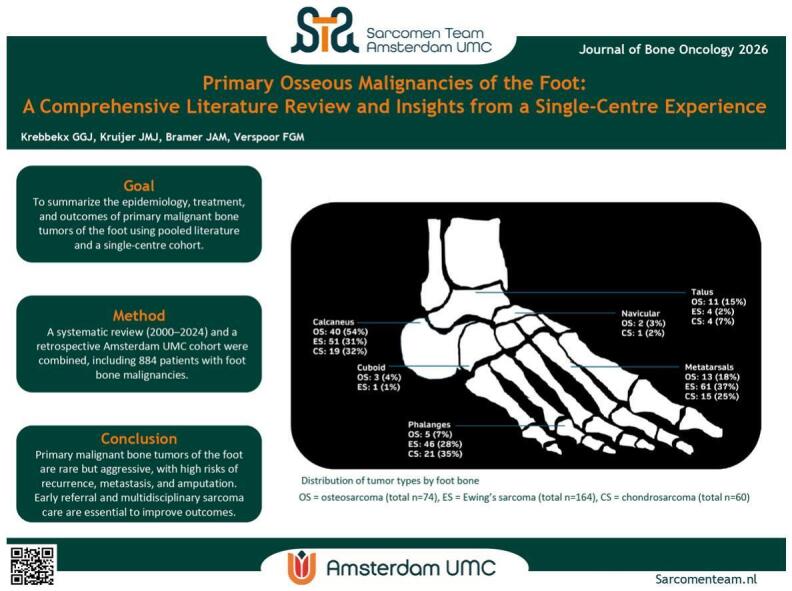

## 1 Introduction

Primary malignant bone tumors of the foot are rare and present distinct diagnostic and therapeutic challenges. Foot tumors account for 3.5–17.2% of all musculoskeletal neoplasms, of which only 5.1–13.9% are primary malignant bone tumors [1], [2], [3], [4]. The most frequent entities are osteosarcoma, Ewing’s sarcoma, and chondrosarcoma, while other malignancies such as fibrosarcoma, epithelioid hemangioendothelioma, angiosarcoma, malignant giant cell tumor, and malignant fibrous histiocytoma are exceptionally uncommon and reported mainly in case studies [1], [3], [5], [6], [7].

Despite the compact anatomy of the foot, which might be expected to facilitate early detection, delayed diagnosis of malignant bone tumors is common [8]. The median interval from symptom onset to diagnosis can be several months, with one series reporting 52 weeks [8], [9], [10]. Contributing factors include the predominance of benign lesions (≈80%), non-specific or mild symptoms, and lack of distinctive clinical features [1], [3], [8], [11]. Consequently, inadvertent excisions occur frequently, increasing the risk of local recurrence and complicating further management [8], [11], [12], [13], [14].

Optimal management of primary malignant bone tumors of the foot requires a multidisciplinary approach. Wide-margin resection is central, and the foot’s anatomy and function often limit limb salvage [13], [15]. Advances in imaging, surgery, and reconstruction have improved outcomes, though below-knee amputation (BKA) remains necessary when margins or function cannot be preserved [12], [13]. Chemotherapy and radiotherapy are key adjuvant therapies, especially for Ewing’s sarcoma and high-grade osteosarcoma [11], [12], [16].

Functional outcome and quality of life are important considerations in the management of foot sarcomas. Recent literature suggests that, when feasible, limb-salvage surgery can yield satisfactory functional results and survival rates, though these must be balanced against the risk of local recurrence [12], [16]. Prognosis depends on tumor type, size, grade, metastasis at diagnosis, and adequacy of surgical margins, with some studies suggesting outcomes comparable or superior to other skeletal sites, potentially due to smaller tumor volumes and less aggressive biological behavior [8], [17].

Research is limited by the rarity of these tumors, with evidence mainly from small retrospective series or registries that lack clinical detail and contain heterogeneous populations, complicating outcome comparisons [3], [5], [18], [19].

Given these challenges, focused studies are needed to provide site-specific data on management and outcomes of primary malignant bone tumors of the foot. This review addresses this gap by synthesizing published evidence and our own data to inform clinical practice and guide future research.

## 2 Methods

### 2.1 Study design overview

This study consists of two components: [1] a retrospective single-centre cohort analysis and [2] a systematic literature review. The methodology was developed in accordance with the STROBE [20] and PRISMA [21] guidelines, and the quality of included studies was assessed using the Joanna Briggs Institute (JBI) Critical Appraisal Checklist for Case Series [22].

### 2.2 Single-Centre cohort

A retrospective cohort study was performed using the MUsculoSkeletal Tumor (MUST)-registry at Amsterdam UMC. All patients diagnosed between May 2013 and May 2024 with histopathologically confirmed primary malignant bone tumors of the foot (excluding other entities than chondrosarcoma, Ewing sarcoma and osteosarcoma) were included, based on WHO criteria [23]. The foot was defined as tarsal bones, metatarsals, and phalanges. Data were collected on demographics, tumor characteristics, symptoms, diagnostic delay, imaging findings, treatment (surgery, radiotherapy, chemotherapy), and oncologic outcomes (local recurrence, metastases, follow-up duration, and status). Tumor size was measured on MRI by a musculoskeletal radiologist, using the longest diameter. Pathology and radiology data were validated via specialist review. Delay in diagnosis was calculated from patient-reported symptom onset to definitive diagnosis. Table 1, Table 2.

**Table 1:** Patient demographics and tumor characteristics single-centre cohort.

Pt	Diagnosis	Age in years	M/F	Location (R/L)	Size in cm on MRI	Symptoms	Delay in months	DD based on imaging
1	Osteosarcoma	20	M	Calcaneus (L)	6.4	Pain, swelling	6	Angiomatous proliferation, EHE, angiosarcoma, GCT
2	Osteosarcoma	20	F	Talus (L)	15	Pain	10	Osteoblastoma, osteosarcoma
3	Ewing’s sarcoma	11	M	MT II (R)	4.5	N/A	9	N/A
4	Ewing’s sarcoma	13	M	Talus (L)	9.0	Pain, swelling	4	N/A
5	Ewing’s sarcoma	14	M	MT V (L)	1.3	Pain, swelling	8	Osteomyelitis, stress fracture
6	Chondrosarcoma	33	F	Phalanx III (L)	2.4	Swelling	120	Enchondroma
7	Chondrosarcoma	35	M	Phalanx IV (L)	1.8	Pain, swelling	55	GCT-B, FTS, myxoid liposarcoma
8	Chondrosarcoma	42	M	Phalanx II (R)	1.5	Pain	513	Enchondroma, ABC
9	Chondrosarcoma	55	M	MT V (R)	4.5	Swelling	0	Chondrosarcoma
10	Chondrosarcoma	42	M	Talus (R)	4.8	Pain, swelling	12	N/A

N/A = data not available, M = male, F = female, R = right foot, L = left foot, MRI = Magnetic Resonance Imaging, DD = differential diagnosis, MT = metatarsal, EHE = epithelioid hemangioepithelioma, GCT-B = giant cell tumor of bone, FTS = fibroma of the tendon sheath, ABC = aneurysmal bone cyst.

**Table 2:** Treatment characteristics and oncologic outcomes single-centre cohort.

Pt	Diagnosis	Surgery	RT	CTx	LR	Metastases	FU-time in months	FU-status
1	Osteosarcoma	ILC, phen. & cement	−	−	After 6 months	−	24	AWD
2	Osteosarcoma	BKA	−	MAP^a^	−	Ossal and soft-tissue	23	DOD
3	Ewing’s sarcoma	AP ray II & os cuneiforme med.	54 Gy	VIDE^a^ & VAC^b^	After 36 months	Pulmonary	134	AWD
4	Ewing’s sarcoma	BKA	−	VIDE^a^ & VAC^b^	−	Pulmonary and ossal	39	DOD
5	Ewing’s sarcoma	AP dig V	−	VIDE^a^ & VAI^b^	−	−	136	NED
6	Chondrosarcoma	ILC	−	−	−	−	50	NED
7	Chondrosarcoma	AP dig IV	−	−	−	−	19	NED
8	Chondrosarcoma	AP dig II	−	−	−	−	55	NED
9	Chondrosarcoma	AP ray V	−	−	−	−	51	NED
10	Chondrosarcoma	ILC, phen.	−	−	After 24 months	Ossal and soft-tissue	237	NED

RT = radiotherapy, CTx = chemotherapy, LR = local recurrence, FU = follow-up, BKA = below-knee amputation, AP = amputation, ILC = intralesional curettage, phen. = phenolization, Gy = Gray, MAP = Methotrexate + Doxorubicin + Cisplatin, V = Vincristine, I = Ifosfamide, D = Doxorubicin, E = Etoposide, A = Actinomycin D, C = Cyclophosphamide, NED = no evidence of disease, AWD = alive with disease, DOD = dead of disease.

a Neoadjuvant chemotherapy.

b Adjuvant chemotherapy.

Potential confounders (such as age, sex, tumor type, and treatment era) were documented. Given the retrospective design, efforts were made to minimize selection and measurement bias by using standardized data sources and definitions. The study was approved by the Amsterdam UMC Institutional Review Board (2025.0109); all data were anonymized prior to analysis.

### 2.3 Systematic literature review

A systematic search of PubMed and Embase was conducted (January 2000–September 2024) using terms for bone malignancies and foot/ankle anatomy (Appendices 1 and 2). Case reports and conference abstracts were excluded. Articles were screened in Rayyan by two independent reviewers [JK & FV], with inclusion criteria: primary osseous malignancies of the foot, English or German language, and > 10 eligible patients (Appendix 3). Reviews, animal/in vitro studies, and other languages were excluded. Reference lists of included articles were screened for additional eligible studies. Additionally, all corresponding authors of the included articles were contacted to request supplementary or unpublished data relevant to our analysis.

### 2.4 Data Extraction and quality Assessment

Data from both the cohort and included studies were extracted on study design, patient and tumor characteristics, treatments, and outcomes. The Joanna Briggs Institute (JBI) Checklist for Case Series [22] was used to assess methodological quality and risk of bias. Appendix 4*.* Extraction and screening were performed independently by two reviewers, with consensus for disagreements.

### 2.5 Data Handling and definitions

Where possible, data were stratified by tumor type. For continuous variables (age, delay in diagnosis, follow-up time), medians were reported as available. Missing data were marked as ‘not available’ (N/A), and data pooled with other factors were marked as ‘together’ (T).

### 2.6 Statistical analysis

Due to the rarity and heterogeneity of these tumors, only descriptive statistics were used. Results are presented as counts with percentages, and, where appropriate, data were pooled and medians with interquartile ranges were calculated using IBM SPSS Statistics (version 28.0, IBM Corp., Armonk, NY, USA). No inferential statistical analyses were performed.

## 3 Results

### 3.1 Single-Centre cohort

At Amsterdam UMC, 17 patients were treated for malignant bone tumors of the foot between May 2013 and May 2024. After excluding three carcinoma metastases and one atypical cartilaginous tumor (ACT), 10 patients (2 osteosarcomas, 3 Ewing’s sarcomas, 5 chondrosarcomas) were analyzed. Median age was 33 years (IQR 17–49), and 4 (31%) were female. The calcaneus was the most affected site (n = 4, 31%). Median tumor size was 4.6 cm (IQR 2.1–6.6), and median diagnostic delay was 12 months (IQR 7–39). Table 1.

Treatment included BKA (2 patients), ray/digit amputation (5), and intralesional curettage (5). Both patients with BKA had talar tumors, developed metastases, and died of disease within 23 and 39 months after diagnosis. Local recurrence occurred in five patients. Median follow-up was 49 months (IQR 24–95). Among all osteosarcoma and Ewing’s sarcoma patients, three of five were alive at last follow-up (median 39 months, IQR 24–135), only one patient was disease-free. All chondrosarcoma patients were alive and disease-free at last follow-up (median 51 months, IQR 35–146). Detailed functional and quality-of-life outcomes were not systematically recorded. Table 2*.*

### 3.2 Literature search and study selection

**Fig. 1:**
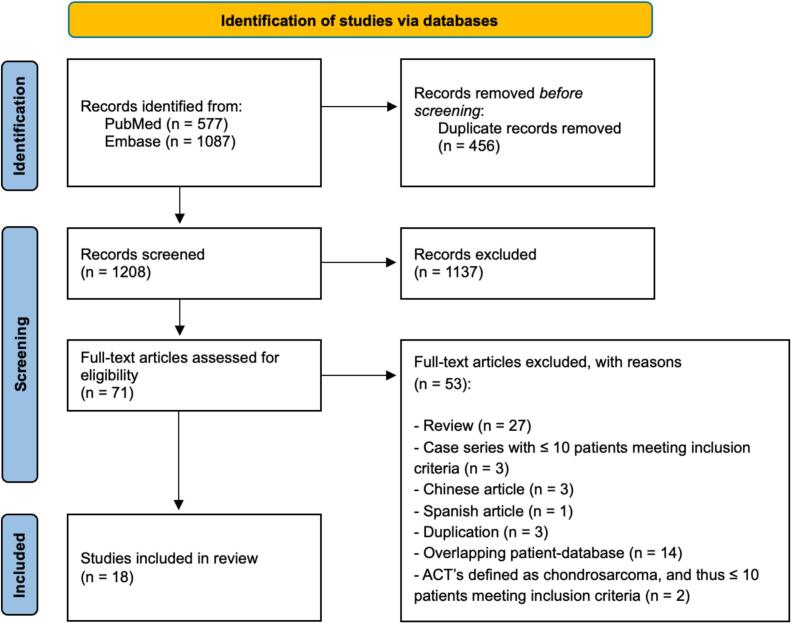
PRISMA flowchart of study selection.

A total of 1,664 articles were identified through the systematic search (Appendices 1 and 2). After removing 456 duplicates, 1,208 articles were screened by title and abstract. Seventy-one full-text articles were assessed for eligibility, of which 18 met the inclusion criteria (Appendix 3). Reasons for exclusion at each stage are detailed in Fig. 1. No additional eligible studies were identified through reference screening. The articles excluded on full text are listed in Appendix 5.

### 3.3 Quality Assessment

Thirteen studies did not clearly report the use of standardized diagnostic criteria, and ten lacked detailed outcome reporting. Overall, risk of bias was moderate to high, primarily due to retrospective design, incomplete reporting, and heterogeneity in case definitions. Missing data were noted for several variables, particularly functional outcomes and long-term follow-up. Appendix 4*.*

### 3.4 Study and patient characteristics

Eighteen retrospective studies published between 2001 and 2024 were included (Table 3). Four studies did not report separate data for foot tumors and were excluded from quantitative synthesis, as indicated by superscripts d and e in Table 3, Table 4, Table 5. In total, 884 patients with primary malignant bone tumors of the foot were analyzed: 231 osteosarcomas, 300 Ewing’s sarcomas, 290 chondrosarcomas/ACTs, and 66 ultra-rare malignancies. Age was reported in 7 studies, while sex was reported in 9 studies. Location was provided in 14 studies, and tumor size was reported in 1 study. Information on symptoms was available in 12 studies, and diagnostic delay was reported in 5 studies. Treatment details, including surgery, radiotherapy, and chemotherapy, were available in 8 studies, while outcomes, including follow-up status and survival, were reported in 8 studies.

**Table 3:** Study design characteristics.

First author	Year	Country	Database	Timeframe of database	Total pts n	Other factors included	Total included pts n	Reported on
Froeb D	2012	Germany	Ewing trial centre, Münster	1991 – 2009	80	Soft-tissue sarcomas, hand	66	Tumor and treatment characteristics and oncologic outcomes
Newman E^a^	2020	USA	2 hospitals	1984–––2015	11	−	11	Tumor and treatment characteristics and oncologic outcomes
Salunke AA	2018	India	1 hospital	2010 – 2017	21	−	21	Tumor and treatment characteristics and oncologic outcomes
Pollandt K^b^	2003	Germany	Hamburg bone tumor registry	N/A	367	Benign bone tumors	37	Tumor characteristics
Toepfer A^b^	2020	Germany	1 hospital	1997 – 2015	409	Benign & soft-tissue tumors, ankle, metastases	20	Tumor characteristics
Karaca MO^c^, ^e^	2022	Turkey	1 hospital	1988 – 2018	80	Soft-tissue sarcomas, ankle, metastases	12	Tumor characteristics
Tsuda Y^c^	2021	UK	1 hospital	1970 – 2018	50	−	50	Tumor and treatment characteristics and oncologic outcomes
Lesensky J^c^	2021	Czech Republic	1 hospital	1976 – 2016	44	Hand	15	Tumor characteristics
Oliveira I^c^, ^d^	2020	UK	1 hospital	2007 – 2019	420	Whole body	35	Tumor characteristics
Ruggieri P^b^	2014	Italy	1 hospital	1990 – 2007	1170	Benign & soft-tissue tumors, metastases, carcinomas	132	Tumor characteristics
Karadeniz S^b^, ^e^	2022	Turkey	1 hospital	2004 – 2021	131	Benign & soft-tissue tumors, ankle	14	Tumor characteristics
Jawad MU^b^	2021	USA	SEER-database	1975 – 2017	514	Soft-tissue sarcomas, hematological malignancies	371	Tumor characteristics
Young PS^a^	2013	UK	SBTR-database	1954 – 2010	57	Benign bone tumors	15	Tumor and treatment characteristics and oncologic outcomes
Baraga JJ	2001	USA	1 hospital & consultation files of 2 doctors	1916 – 1999	43	Hand	32	Tumor and treatment characteristics and oncologic outcomes
Schuster AJ	2018	Germany, Austria, Switzerland	COSS-database (14 hospitals)	1980 – 2016	23	−	23	Tumor and treatment characteristics and oncologic outcomes
Brotzmann M^a^	2013	Switzerland	BTRC-database	1969 – 2008	32	−	32	Tumor characteristics
Bakotic B^c^	2001	USA	1 hospital	1985 – 2000	150	Benign bone tumors, hematological malignancies, metastases	57	Tumor characteristics
Berger M^d^	2013	Italy	AIEOP-database & ISG-database (11 hospitals)	1980 – 2009	112	Hand, craniofacial, mobile spine	37	Tumor characteristics
Amsterdam UMC^a^	2024	Netherlands	1 hospital	2013 – 2024	10	−	10	Tumor and treatment characteristics and oncologic outcomes

Pts = patients, N= Number, N/A = data not available, SEER = Surveillance, Epidemiology, and End Results, SBTR = Scottish Bone Tumour Registry, COSS = Cooperative German-Austrian-Swiss Osteosarcoma Study Group, BTRC = Bone Tumour Reference Center, AIEOP = Association of Pediatric Hematology and Oncology, ISG = Italian Sarcoma Group.

a Articles without ACTs, or ACTs are excluded from the results.

b Articles that don’t mention whether a proportion of the chondrosarcomas are ACTs.

c Articles where a proportion of the chondrosarcomas are ACTs, but results are presented together.

d Results of patients with malignancy in hand and foot are pooled.

e Results of patients with malignancy in foot and ankle are pooled.

**Table 4:** Patient demographics and tumor characteristics.

Study	Tumor type	Pts n(%)	Age med(IQR)	Male %	Anatomically most affected site n(%)		Delay in months med(IQR)
Froeb D et al. (2012)	Ewing’s sarcoma	66(100)	T	T	Metatarsals	33(50)	N/A
Newman E et al. (2020)^a^	Osteosarcoma	5(46)	39(17–52)	60%	Calcaneus	5(100)	5(4–10)
	Ewing’s sarcoma	2(18)	14(range 9–19)	50%	Calcaneus	2(100)	36
	Chondrosarcoma	1(9)	21	100%	Calcaneus	1(100)	3
	Other^1^	3(27)	55(range 20–61)	100%	Calcaneus	3(100)	7(range 2–12)
Salunke AA et al. (2018)	Osteosarcoma	14(67)	22(18–28)	86%	Calcaneus	11(79)	9(8–9)
	Ewing’s sarcoma	7(33)	20(12–22)	86%	Calcaneus	6(86)	6(6–12)
Pollandt K et al. (2003)^b^	Osteosarcoma	10(27)	T	N/A	Calcaneus	5(50)	N/A
	Ewing’s sarcoma	8(22)	T	N/A	Metatarsals	4(50)	N/A
	Chondrosarcoma	19(51)	T	N/A	Calcaneus	9(47)	N/A
Toepfer A et al. (2020)^b^	Osteosarcoma	2(10)	T	T	Midfoot	1(50)	N/A
					Hindfoot	1(50)	
	Ewing’s sarcoma	4(20)	T	100%	Midfoot	2(50)	N/A
					Hindfoot	2(50)	
	Chondrosarcoma	13(65)	T	T	Hindfoot	6(46)	N/A
	Other^2^	1(5)	T	100%	Forefoot	1(100)	N/A
Karaca MO et al. (2022)^c,e^	Osteosarcoma	5(42)	T	N/A	T	T	N/A
	Ewing’s sarcoma	3(25)	T	N/A	T	T	N/A
	Chondrosarcoma	3(25)	T	N/A	T	T	N/A
	ACT	1(8)					
Tsuda Y et al. (2021)^c^	Osteosarcoma	13(26)	35(19–55)	62%	Calcaneus	6(46)	N/A
	Ewing’s sarcoma	14(28)	14(12–17)	50%	Phalangen	6(43)	N/A
	Chondrosarcoma	15(30)	55(45–72)	52%	Metatarsals	10(43)	N/A
	ACT	8(16)					
Lesensky J et al. (2021)^c^	Chondrosarcoma	15(100)	T	T	Metatarsals	N/A	T
Oliveira I et al. (2020)^c,d^	Chondrosarcoma	35(100)	T	T	Phalangen	N/A	N/A
Ruggieri P et al. (2014)^b^	Osteosarcoma	24(18)	T	N/A	Hindfoot	20(83)	N/A
	Ewing’s sarcoma	44(33)	T	N/A	Hindfoot	26(59)	N/A
	Chondrosarcoma	29(22)	T	N/A	Hindfoot	14(48)	N/A
	Other^3^	35(27)	T	N/A	Hindfoot	21(60)	N/A
Karadeniz S et al. (2022)^b,e^	Osteosarcoma	2(14)	T	T	N/A	T	N/A
	Ewing’s sarcoma	10(71)	T	T	N/A	T	N/A
	Chondrosarcoma	2(14)	T	T	N/A	T	N/A
Jawad MU et al. (2021)^b^	Osteosarcoma	108(29)	T	T	N/A	T	N/A
	Ewing’s sarcoma	93(25)	T	T	N/A	T	N/A
	Chondrosarcoma	160(43)	T	T	N/A	T	N/A
	Other^4^	10(3)	T	T	N/A	T	N/A
Young PS et al. (2013)^a^	Osteosarcoma	7(44)	44(20–75)	57%	Calcaneus	6(86)	T
	Ewing’s sarcoma	2(13)	25(range 17–32)	100%	Calcaneus	2(100)	
	Chondrosarcoma	7(44)	41(19–64)	86%	Calcaneus	5(71)	
Baraga JJ et al. (2001)	Ewing’s sarcoma	32(100)	T	T	Metatarsals	14(44)	N/A
Schuster AJ et al. (2018)	Osteosarcoma	23(100)	32(13–44)	57%	Calcaneus	10(43)	5(range 1–64)
Brotzmann M et al. (2013)^a^	Osteosarcoma	9(39)	45(31–54)	22%	Metatarsals	5(56)	15(3–19)
	Ewing’s sarcoma	8(35)	17(12–19)	75%	Metatarsals	5(63)	18(5–26)
	Chondrosarcoma	6(26)	65(22–69)	67%	Phalangen	3(50)	10(6–12)
Bakotic B et al. (2001)^c^	Osteosarcoma	14(25)	T	50%	T	T	N/A
	Ewing’s sarcoma	17(30)	T	59%	T	T	N/A
	Chondrosarcoma	7(12)	T	67%	T	T	N/A
	ACT	5(9)					
	Other^5^	14(25)	T	57%	T	T	N/A
Berger M et al. (2013)^d^	Ewing’s sarcoma	37(100)	T	T	N/A	T	T
Amsterdam UMC (2024)^a^	Osteosarcoma	2(15)	20	50%	Calcaneus	1(50)	8(range 6–10)
					Talus	1(50)	
	Ewing’s sarcoma	3(23)	13(range 11–14)	100%	Metatarsals	2(67)	8(range 4–9)
	Chondrosarcoma	5(38)	42(34–49)	80%	Phalangen	3(60)	55(6–317)

T = presented together with other factors, N/A = data not available, med = mediaan, IQR = interquartile range.

^a^Articles without ACTs, or ACTs are excluded from the results.

^b^Articles that don’t mention whether a proportion of the chondrosarcomas are ACTs.

^c^Articles where a proportion of the chondrosarcomas are ACTs, but results are presented together.

^d^Results of patients with malignancy in hand and foot are pooled.

^e^Results of patients with malignancy in foot and ankle are pooled.

^1^Fibrosarcoma (n=1), angiosarcoma (n=1), EHE (n=1).

^2^Fibrosarcoma (n=1).

^3^Fibrosarcoma (n=9), EHE (n=26).

^4^Malignant giant cell tumor of bone (n=10).

^5^Fibrosarcoma (n=2), angiosarcoma (n=1), EHE (n=8), MFH (n=2), NOS (n=1).

^6^Myoepithelioma (n=1), Kaposi sarcoma (n=1), malignant giant cell tumor of bone (n=1).

**Table 5:** Treatment characteristics and oncologic outcomes.

Study	Tumor type	Pts n	Surgery n	RT n(%)	CTx n(%)	LR n(%)	Mets. n(%)	FU-time in months med(IQR)	FU-status
Froeb D et al. (2012)	Ewing’s sarcoma	66	N/A	T	66(100)	N/A	T	N/A	T
Newman E et al. (2020)^a^	Osteosarcoma	5	BKA5	0	5(100)	0	3(60)	65(21–241)	NED 3, AWD 1, DOD 1
	Ewing’s sarcoma	2	BKA 1, ILC 1	1(50)	2(100)	0	0	162(range 124–199)	NED 2
	Chondrosarcoma	1	BKA 1	0	1(100)	0	0	24	DOD 1
	Other^1^	3	BKA 2, ILC 1	1(33)	0	0	3(100)	79(range 32–203)	NED 1, DOD 2
Salunke AA et al. (2018)	Osteosarcoma	14	BKA 14	N/A	14(100)	8(57)	2(14)	37(34–47)	AWD 5, DOD 9
	Ewing’s sarcoma	7	BKA 7	N/A	7(100)	2(29)	4(57)	43(32–66)	AWD 4, DOD 3
Tsuda Y et al. (2021)^c^	Osteosarcoma	13	BKA 8, AP ray 3, ILC 2	0	4(31)	1(8)	3(23)	49(27–66)	NED 10, AWD 1, DOD 2
	Ewing’s sarcoma	14	BKA 7, AP ray 6, AP dig 1	2(14)	14(100)	0	5(36)	74(25–105)	NED 9, AWD 2, DOD 2, DOOD 1
	Chondrosarcoma	15	BKA 6, AP ray 9^f^	0	0	2(9)	3(13)	75(47–119)	NED 19, AWD 1, DOOD 3
	ACT	8							
Young PS et al. (2013)^a^	Osteosarcoma	7	BKA 6, none 1	0	2(29)	0	2(29)	108(24–168)	NED 3, DOD 3, DOOD 1
	Ewing’s sarcoma	2	BKA 2	2(100)	1(50)	0	2(100)	42(range 12–72)	DOD 2
	Chondrosarcoma	7	BKA 3, RES 1, ILC 3	0	0	0	1(14)	84(18–96)	NED 3, DOD 1, DOOD 3
Baraga JJ et al. (2001)	Ewing’s sarcoma	32	N/A	N/A	N/A	N/A	N/A	N/A	N/A
Schuster AJ et al. (2018)	Osteosarcoma	23	BKA 9, AP part foot 4, AP ray 2, AP dig 1, RES 5, ILC 2	N/A	22(96)	N/A	3(13)	48(17–79)	NED 15, AWD 1, DOD 6, DOOD 1
Brotzmann M et al. (2013)^a^	Osteosarcoma	9	BKA 7, RES 1, ILC 1	N/A	N/A	1(11)	5(56)	102(30–174)	NED 5, DOD 4
	Ewing’s sarcoma	8	BKA 5, RES 2, ILC 1	N/A	8(100)	2(25)	7(88)	68(44–110)	NED 2, AWD 1, DOD 5
	Chondrosarcoma	6	BKA 4, ILC 2	N/A	N/A	2(33)	2(33)	96(83–147)	NED 3, DOD 2, DOOD 1
Amsterdam UMC (2024)^a^	Osteosarcoma	2	BKA 1, ILC 1	0	1(50)	1(50)	1(50)	24(range 23–24)	AWD 1, DOD 1
	Ewing’s sarcoma	3	BKA 1, AP ray 1, AP dig 1	1(33)	3(100)	1(33)	2(67)	134(range 39–136)	NED 1, AWD 1, DOD 1
	Chondrosarcoma	5	AP ray 1, AP dig 2, ILC 2	0	0	1(20)	1(20)	51(35–146)	NED 5

T = presented together with other factors, N/A = data not available, RT = radiotherapy, CTx = chemotherapy, LR = local recurrence, FU = follow-up, med = mediaan, IQR = interquartile range, BKA = below-knee amputation, AP = amputation, RES = wide or marginal resection, ILC = intralesional curettage, NED = no evidence of disease, AWD = alive with disease, DOD = dead of disease, DOOD = dead of other disease.

^a^Articles without ACTs, or ACTs are excluded from the results.

^b^Articles that don’t mention whether a proportion of the chondrosarcomas are ACTs .

^c^Articles where a proportion of the chondrosarcomas are ACTs, but results are presented together.

^d^Results of patients with malignancy in hand and foot are pooled .

^e^Results of patients with malignancy in foot and ankle are pooled .

^f^ACTs excluded from surgery data.

^1^Fibrosarcoma (n = 1), angiosarcoma (n = 1), EHE (n = 1).

^2^Fibrosarcoma (n = 1).

^3^Fibrosarcoma (n = 9), EHE (n = 26).

^4^Malignant giant cell tumor bone (n = 10).

^5^Fibrosarcoma (n = 2), angiosarcoma (n = 1), EHE (n = 8), MFH (n = 2), NOS (n = 1).

^6^Myoepithelioma (n = 1), Kaposi sarcoma (n = 1), malignant giant cell tumor of bone (n = 1).

**Fig. 2:**
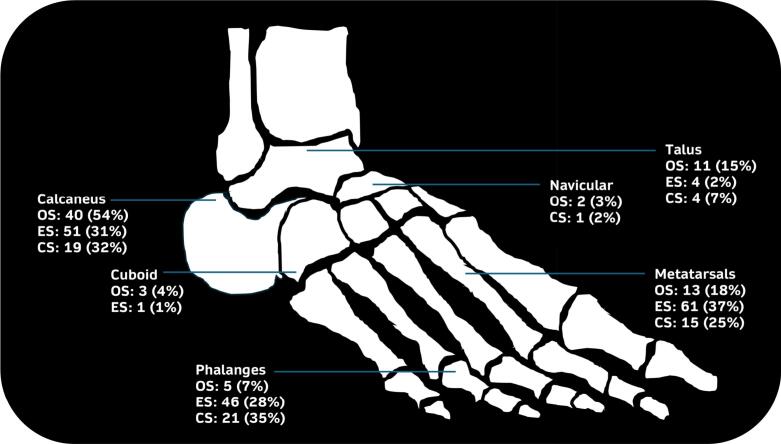
Anatomical distribution of primary malignant bone tumors of the foot. *The figure includes only patients with osteosarcoma, Ewing’s sarcoma, and chondrosarcoma (n = 298).* OS = osteosarcoma (total n = 74), ES = Ewing’s sarcoma (total n = 164), CS = chondrosarcoma (total n = 60).

Median follow-up among patients contributing outcome data ranged from 24 to 162 months across the included studies. Ewing’s sarcoma patients were youngest (n = 22, median 17 years[IQR 12–20]), followed by osteosarcoma (n = 60, median 29 years[IQR 19–45]), chondrosarcoma (n = 19, median 42 years[IQR 22–64]), and ultra-rare malignancies (n = 6, median 56 years[IQR 30–61]). The majority were male (137/219, 63%). The calcaneus was the most frequent site (110/298, 37%), followed by metatarsals (89/298, 30%) and phalanges (72/298, 24%). Among osteosarcoma patients, the calcaneus was the most frequently affected site (40/74, 54%). Ewing’s sarcomas occurred most often in the metatarsals (61/164, 37%), while chondrosarcomas were most commonly found in the phalanges (21/60, 35%). Median largest tumor size on MRI was 4.6 cm (IQR 2.1–6.6), largest in osteosarcoma with a median volume of 31 cm^3^ (IQR 9–60). Fig. 2*,* Table 4*.*

### 3.5 Clinical Presentation, Diagnosis, and delay

Pain (51/59, 86%) and swelling (40/59, 68%) were the most common presenting symptoms. Overall (n = 62) the median diagnostic delay was 9 months (IQR 6–14), about similar by tumor type. Table 4*.*

### 3.6 Treatment Modalities

Overall, BKA was the most common surgical procedure, performed in 61% (89/147) of cases. Among osteosarcoma patients, BKA was performed in 69% (50/72), en-bloc resections (including Chopart, ray, and digit amputations, and wide or marginal resections) in 22% (16/72), and intralesional curettage (ILC) in 8% (6/72). For Ewing’s sarcoma, BKA was performed in 64% (23/36), en-bloc resection in 31% (11/36), and ILC in 6% (2/36). Among chondrosarcoma patients (all grade 2 or higher), BKA was performed in 41% (14/34), en-bloc resection in 38% (13/34), and ILC in 21% (6/34). Table 5.

Adjuvant chemotherapy was administered in most Ewing’s sarcomas (93/94 99%) and high-grade osteosarcoma cases (48/64 75%), radiotherapy was used in 6/21 (29%) patients with Ewing sarcomas, and 0/27 (0%) patients with osteosarcomas. Table 5.

### 3.7 Oncologic outcomes

Local recurrence occurred in 12–33% of cases, with higher rates in patients with positive margins or after intralesional procedures. Local recurrence rates were 19% for osteosarcoma (14/73), 14% for Ewing’s sarcoma (5/36), and 12% for chondrosarcoma (5/42). Metastatic disease developed most often in Ewing’s sarcoma patients (20/36, 56%), followed by osteosarcoma (25/73, 34%) and chondrosarcoma (8/42, 19%).

Survival at last follow-up, 43% of osteosarcoma patients (26/60) were alive without disease, 13% (8/60) were alive with disease, and 40% (24/60) had died of disease (median follow-up 47 months, IQR 24–97). For Ewing’s sarcoma (n = 22, median follow-up 66 months, IQR 60–70), 23% (5/22) were disease-free, 27% (6/22) were alive with disease, and 50% (11/22) had died. Among chondrosarcoma patients (n = 19, median follow-up 82 months, IQR 50–96), 58% (11/19) were disease-free and 21% (4/19) had died of disease. Death from other causes occurred in 3% of osteosarcoma cases (2/60) and 21% of chondrosarcoma cases (4/19). Long-term (5- or 10-year) survival data were inconsistently reported in most studies. Table 5*.*

Functional outcomes and quality of life were rarely reported. Where available, limb-salvage patients generally had better functional scores than amputees, but some experienced significant morbidity due to reconstructive failure or chronic pain. Data on return to work, activities of daily living, and patient-reported outcomes were largely missing.

### 3.8 Missing data

Data on surgical margins, adjuvant therapy, functional outcomes, and long-term survival or quality of life were not consistently available.

## 4 Discussion

This systematic review and single-centre cohort analysis confirm that osteosarcoma, Ewing’s sarcoma, and chondrosarcoma are the most common primary malignant bone tumors of the foot, with the calcaneus, metatarsals, and phalanges being the most frequently affected sites for each respective tumor type. Most patients presented with pain and swelling, and BKA remained the most common surgical procedure, especially for hindfoot tumors. Local recurrence rates were highest in osteosarcoma, while Ewing’s sarcoma patients most frequently developed metastatic disease. Disease-specific mortality at last follow-up was substantial across all tumor types, underscoring the aggressive nature of these malignancies even in the foot.

The outcomes for primary malignant bone tumors of the foot appear to differ in some respects from those in other skeletal locations [8]. Notably, chondrosarcoma of the foot may have a more favorable prognosis compared to chondrosarcoma of the pelvis or long bones, possibly due to earlier detection or intrinsic biological differences [8]. An explanation is the potential contamination of reported series with ACTs, which behave less aggressively and may therefore account for the more favorable oncologic outcomes of foot chondrosarcomas compared to other sites [8], [17], [24]. Conversely, osteosarcoma and Ewing’s sarcoma of the foot continue to have significant risks of recurrence and metastasis, similar to their counterparts in other locations [8], [11], [17]. However, direct comparative studies are limited, and further research is needed to clarify whether anatomical site independently influences prognosis.

Diagnostic delay remains a significant challenge, with a median delay of 9 months observed across tumor types. Delays were longest for chondrosarcom [9], [25], likely reflecting its indolent presentation. Prolonged time to diagnosis may contribute to larger tumor size, more advanced stage at presentation, and the need for more extensive surgery, potentially worsening outcomes [25]. Efforts to raise awareness among primary care providers and improve access to specialist evaluation are critical for earlier diagnosis and improved prognosis [26], [27].

The role of adjuvant chemotherapy and radiotherapy varies by tumor type. Most patients with Ewing’s sarcoma and high-grade osteosarcoma received chemotherapy, which is standard of care and associated with improved survival [6], [28]. Radiotherapy was used selectively, primarily for Ewing’s sarcoma or unresectable disease [28], [29]. Standardized reporting on effects and dose of adjuvant treatment is needed, as the effectiveness of adjuvant treatment in foot tumors remains unclear due to limited and heterogeneous data.

Centralized care in specialized sarcoma centers with multidisciplinary teams is important [25], [27], as inadvertent or unplanned surgeries, often resulting from initial misdiagnosis, were associated with higher rates of amputation, recurrences, and mortality [14], [25]. This may be partly attributed to the relatively high incidence of benign lesions, which may result in diagnostic inaccuracies among physicians lacking specific expertise in bone tumors[27] . Early referral, appropriate imaging, and biopsy prior to definitive surgery are essential to optimize outcomes and minimize the risk of inadequate initial management [25], [27].

A major gap in the current literature is the lack of functional outcome and quality of life data [6], [28]. While limb-sparing procedures are increasingly attempted, BKA remains common, particularly for extensive or misdiagnosed tumors [14], [25]. The functional and psychosocial impact of these interventions on mobility, independence, and return to daily activities is underreported. Future studies should prioritize patient-reported outcome measures and long-term functional follow-up to inform shared decision-making and rehabilitation strategies.

Recent advances in molecular diagnostics, particularly for Ewing’s sarcoma and rare subtypes, offer opportunities for more precise diagnosis, risk stratification, and targeted therapy [30]. Incorporating molecular and genetic profiling into future research and clinical practice may improve individualized treatment and prognostication.

The current literature lacks data on the impact of socioeconomic status, ethnicity, and healthcare access on diagnostic delay, treatment, and outcomes. These factors may influence presentation and prognosis and should be addressed in future studies to ensure equitable care.

This review is limited by the rarity of primary malignant bone tumors in the foot, resulting in small, retrospective case series with heterogeneous populations and reporting [6], [8], [31]. Many studies lacked standardized definitions of anatomical sites, tumor grades, and inclusion criteria, introducing potential selection and detection bias. Publication and language bias may also be present, as only English and German articles were included. The lack of data on surgical margins, function and quality of life, as well as inconsistent reporting on adjuvant therapy and long-term survival, further limits the strength of our conclusions. Excluding the five studies with fewer than 10 cases may have limited the inclusion of valuable data. However, including such small studies could have introduced significant variability and bias, ultimately compromising the robustness and reliability of the review.

### 4.1 Future Directions

To advance the field, future research should focus on:

• Prospective, multicenter registries with standardized definitions and comprehensive data collection, including functional and patient-reported outcomes [6], [25], [27]
• Comparative studies evaluating outcomes by anatomical site and treatment modality [14]
• Integration of molecular and genetic profiling into routine diagnosis and research [30], [32]
• Investigation of the impact of diagnostic delay and socioeconomic factors on outcomes [25]
• Development of clinical guidelines emphasizing early referral, multidisciplinary management, and avoidance of unplanned surgery [14], [25], [27].

### 4.2 Clinical recommendations

Clinicians should maintain a high index of suspicion for malignancy in patients with persistent foot pain or swelling, especially when imaging is inconclusive [27]. Early referral to specialized centers, careful biopsy, and multidisciplinary planning are essential to optimize outcomes. Avoidance of unplanned or inadequate surgery is critical, as it is associated with worse prognosis [14], [25], [27].

## 5 Conclusion

In summary, while primary malignant bone tumors of the foot are rare, they present unique diagnostic and therapeutic challenges. Improved awareness, standardized management, and collaborative research are needed to enhance outcomes and quality of life for affected patients.

## CRediT authorship contribution statement

**Juul M.J. Kruijer:** Writing – original draft, Visualization, Project administration, Methodology, Investigation, Formal analysis, Data curation, Conceptualization. **Gitte G Krebbekx:** Writing – review & editing, Writing – original draft, Supervision, Methodology, Investigation, Funding acquisition, Formal analysis, Data curation, Conceptualization. **Jos AM Bramer:** Writing – review & editing, Supervision, Project administration, Investigation, Conceptualization. **Floortje G.M. Verspoor:** Writing – review & editing, Writing – original draft, Visualization, Validation, Supervision, Project administration, Methodology, Investigation, Formal analysis, Data curation, Conceptualization.

## Declaration of competing interest

The authors declare that they have no known competing financial interests or personal relationships that could have appeared to influence the work reported in this paper.
