# Supplementary material for: Primary osseous malignancies of the foot: a comprehensive literature review and insights from a single-centre experience
Source: J Bone Oncol. 2026 Mar 12;58:100755. doi: 10.1016/j.jbo.2026.100755 (PMC13015207; doi:10.1016/j.jbo.2026.100755)
Supplement: Supplementary Data 1 [file mmc1.docx]

# Appendices

Appendix 1. Pubmed search strategy

Appendix 2. Embase search strategy

Appendix 3. Inclusion and exclusion criteria

Appendix 4. Joanna Briggs Institute (JBI) Critical Appraisal Checklist for Case Series: results

Appendix 5. Articles excluded on full text

# Appendix 1. PubMed search strategy

| Search | |
| --- | --- |
| Bone cancer or osteosarcoma or Ewing sarcoma or chondrosarcoma | (((“bone and bones”[MeSH] OR (“bone*”[TIAB] OR “bone tissue”[TIAB])) AND (“neoplasm*, malignant”[TIAB] OR “malignant”[TIAB] OR “malignant neoplasm*”[TIAB] OR “cancer*”[TIAB])) OR (“bone cancer”[TIAB] OR “cancer of bone”[TIAB] OR “cancer of the bone”[TIAB]) OR (“osteosarcoma”[MeSH] OR (“osteosarcoma*”[TIAB] OR “osteogenic sarcoma*“[TIAB] OR “osteosarcoma tumor“[TIAB] OR “tumor, osteosarcoma*“[TIAB] OR “sarcoma*, osteogenic“[TIAB])) OR (“sarcoma, ewing”[MeSH] OR (“ewing* sarcoma*“[TIAB] OR “ewing* tumor“[TIAB] OR “tumor*, ewing*“[TIAB] OR “sarcoma*, ewing*“[TIAB])) OR (“chondrosarcoma”[MeSH] OR (“chondrosarcoma*”[TIAB]))) |
|  | **AND** |
| Foot or ankle or foot bones | ((“foot”[MeSH] OR “foot bones”[MeSH] OR (“foot”[TIAB] OR “bone*, foot”[TIAB] OR “bones of feet”[TIAB] OR “feet bone*”[TIAB] OR “foot bone*”[TIAB] OR “feet”[TIAB])) OR (“ankle”[MeSH] OR (“ankle*”[TIAB] OR “ankle bone*”[TIAB] OR “bone*, ankle”[TIAB])) OR (“tarsal bones”[MeSH] OR (“bone*, tarsal”[TIAB] OR “tarsal bone*”[TIAB] OR “tarsus”[TIAB] OR “regio tarsalis”[TIAB])) OR (“calcaneus”[MeSH] OR (“calcaneus”[TIAB] OR “calcaneus bone*”[TIAB])) OR (“talus”[MeSH] OR (“talus”[TIAB] OR “talus bone*”[TIAB] OR “astragalus bone*”[TIAB] OR “bone*, astragalus”[TIAB])) OR (“cuboid bone*”[TIAB] OR “bone*, cuboid”[TIAB] OR “os cuboideum”[TIAB] OR “cuboid”[TIAB]) OR (“navicular bone*”[TIAB] OR “os naviculare*”[TIAB] OR “naviculare*, os”[TIAB] OR “navicular*”[TIAB]) OR (“cuneiform bone*”[TIAB] OR “bone*, cuneiform”[TIAB] OR “ossa cuneiformia”[TIAB] OR “os cuneiforme*”[TIAB]) OR (“metatarsal bones”[MeSH] OR (“bone*, metatarsal”[TIAB] OR “metatarsal bone*”[TIAB] OR “metatarsal*”[TIAB])) OR (“toe phalanges”[MeSH] OR (“toe phalanges”[TIAB] OR “phalanges of toe”[TIAB:~0] OR “phalanges of toes”[TIAB] OR “phalanges, toe”[TIAB:~0] OR “phalanges, toes”[TIAB:~0] OR “bone* of toes”[TIAB] OR “toe bone*”[TIAB] OR “bone*, toe”[TIAB])) OR (“os trigonum”[TIAB] OR “os peroneum”[TIAB] OR “ossa sesamoidea”[TIAB] OR “os sesamoideum ”[TIAB] OR “sesamoid bone*”[TIAB])) |
|  | **NOT** |
| Filter | “case reports”[pt] |
|  | **AND** |
| Filter | (2000:2024[pdat]) |

# Appendix 2. Embase search strategy

| Search | |
| --- | --- |
| Bone cancer or osteosarcoma or Ewing sarcoma or chondrosarcoma | ('bone cancer':ti,ab,kw OR 'cancer of bone':ti,ab,kw OR 'cancer of the bone':ti,ab,kw OR (('bone'/mj OR 'bones'/mj OR 'bone*':ti,ab,kw OR 'bone tissue':ti,ab,kw) AND ('neoplasm*, malignant':ti,ab,kw OR 'malignant':ti,ab,kw OR 'malignant neoplasm*':ti,ab,kw OR 'cancer*':ti,ab,kw)) OR 'osteosarcoma'/mj OR 'osteosarcoma':ti,ab,kw OR 'osteosarcoma*':ti,ab,kw OR 'osteogenic sarcoma*':ti,ab,kw OR 'osteosarcoma tumor':ti,ab,kw OR 'tumor, osteosarcoma*':ti,ab,kw OR 'sarcoma*, osteogenic':ti,ab,kw OR 'ewing sarcoma'/mj OR 'ewing sarcoma':ti,ab,kw OR 'ewing* sarcoma*':ti,ab,kw OR 'ewing* tumor':ti,ab,kw OR 'tumor*, ewing*':ti,ab,kw OR 'sarcoma*, ewing*':ti,ab,kw OR 'chondrosarcoma'/mj OR 'chondrosarcoma*':ti,ab,kw) |
|  | **AND** |
| Foot or ankle or foot bones | ('foot'/mj OR 'foot':ti,ab,kw OR 'foot bone'/mj OR 'foot bone':ti,ab,kw OR 'bone*, foot':ti,ab,kw OR 'bones of feet':ti,ab,kw OR 'feet bone*':ti,ab,kw OR 'foot bone*':ti,ab,kw OR 'feet':ti,ab,kw OR 'ankle'/mj OR 'ankle*':ti,ab,kw OR 'ankle bone*':ti,ab,kw OR 'bone*, ankle':ti,ab,kw OR 'tarsal bone'/mj OR 'bone*, tarsal':ti,ab,kw OR 'tarsal bone*':ti,ab,kw OR 'tarsus':ti,ab,kw OR 'regio tarsalis':ti,ab,kw OR 'calcaneus'/mj OR 'calcaneus':ti,ab,kw OR 'calcaneus bone*':ti,ab,kw OR 'talus'/mj OR 'talus':ti,ab,kw OR 'talus bone*':ti,ab,kw OR 'astragalus bone*':ti,ab,kw OR 'bone*, astragalus':ti,ab,kw OR 'cuboid bone*':ti,ab,kw OR 'bone*, cuboid':ti,ab,kw OR 'os cuboideum':ti,ab,kw OR 'cuboid':ti,ab,kw OR 'navicular bone*':ti,ab,kw OR 'os naviculare*':ti,ab,kw OR 'naviculare*, os':ti,ab,kw OR 'navicular*':ti,ab,kw OR 'cuneiform bone*':ti,ab,kw OR 'bone*, cuneiform':ti,ab,kw OR 'ossa cuneiformia':ti,ab,kw OR 'os cuneiforme*':ti,ab,kw OR 'metatarsal bone'/mj OR 'bone*, metatarsal':ti,ab,kw OR 'metatarsal bone*':ti,ab,kw OR 'metatarsal*':ti,ab,kw OR 'toe phalanx'/mj OR 'toe phalanx':ti,ab,kw OR 'toe phalanges':ti,ab,kw OR 'phalanges of toe':ti,ab,kw OR 'phalanges of toes':ti,ab,kw OR 'phalanges, toe':ti,ab,kw OR 'phalanges, toes':ti,ab,kw OR 'bone* of toes':ti,ab,kw OR 'toe bone*':ti,ab,kw OR 'bone*, toe':ti,ab,kw OR 'os trigonum':ti,ab,kw OR 'os peroneum':ti,ab,kw OR 'ossa sesamoidea':ti,ab,kw OR 'os sesamoideum':ti,ab,kw OR 'sesamoid bone*':ti,ab,kw) |
|  | **AND** |
| Filter | ('article'/it OR 'review'/it) |
|  | **AND** |
| Filter | [2000-2024]/py |

# Appendix 3: Inclusion and exclusion criteria

| Inclusion criteria | Exclusion criteria |
| --- | --- |
| Patients with primary osseous malignancies of the foot | Study cohorts with ≤ 10 eligible patients |
| English articles | Article reviews |
| German articles | Animal research & in vitro research |
|  | Other languages than English of German |

# Appendix 4. Joanna Briggs Institute (JBI) Critical Appraisal Checklist for Case Series: results

Yes No Unclear

N.A. = Not applicable

**Questions 1 – 5**

| Study | Inclusion criteria | Standard measurement | Valid identification | Consecutive inclusion | Complete inclusion |
| --- | --- | --- | --- | --- | --- |
| Froeb D et al. |  |  |  |  |  |
| Newman E et al. |  |  |  |  |  |
| Salunke AA et al. |  |  |  |  |  |
| Pollandt K et al. |  |  |  |  |  |
| Toepfer A et al. |  |  |  |  |  |
| Karaca MO et al. |  |  |  |  |  |
| Tsuda Y et al. |  |  |  |  |  |
| Lesensky J et al. |  |  |  |  |  |
| Oliveira I et al. |  |  |  |  |  |
| Ruggieri P et al. |  |  |  |  |  |
| Karadeniz S et al. |  |  |  |  |  |
| Jawad MU et al. |  |  |  |  |  |
| Young PS et al. |  |  |  |  |  |
| Baraga JJ et al. |  |  |  |  |  |
| Schuster AJ et al. |  |  |  |  |  |
| Brotzmann M et al. |  |  |  |  |  |
| Bakotic B et al. |  |  |  |  |  |
| Berger M et al. |  |  |  |  |  |

**Questions 6 – 10**

| Study | Demographic information | Clinical information | Outcomes / follow-up | Demographics of sites/clinics | Statistical analysis |
| --- | --- | --- | --- | --- | --- |
| Froeb D et al. |  |  |  |  |  |
| Newman E et al. |  |  |  |  | N.A. |
| Salunke AA et al. |  |  |  |  |  |
| Pollandt K et al. |  |  |  |  | N.A. |
| Toepfer A et al. |  |  |  |  |  |
| Karaca MO et al. |  |  |  |  |  |
| Tsuda Y et al. |  |  |  |  |  |
| Lesensky J et al. |  |  |  |  |  |
| Oliveira I et al. |  |  |  |  |  |
| Ruggieri P et al. |  |  |  |  |  |
| Karadeniz S et al. |  |  |  |  |  |
| Jawad MU et al. |  |  |  |  |  |
| Young PS et al. |  |  |  |  |  |
| Baraga JJ et al. |  |  |  |  |  |
| Schuster AJ et al. |  |  |  |  |  |
| Brotzmann M et al. |  |  |  |  |  |
| Bakotic B et al. |  |  |  |  |  |
| Berger M et al. |  |  |  |  |  |

# Appendix 5. Articles excluded on full text

| Study | Year | Journal | Reason for exclusion |
| --- | --- | --- | --- |
| Lichte P et al. | 2022 | Fuß & Sprunggelenk | Review |
| Farei-Campagna JM et al. | 2023 | Fuß & Sprunggelenk | Review |
| Temple HT | 2002 | Curr Opin Orthop | Review |
| Maldjian C et al. | 2001 | Magn Reson Imaging Clin North Am | Review |
| Temple EW et al. | 2021 | Clin Podiatr Med Surg | Review |
| Rammelt S et al. | 2023 | Foot Ankle Surg | Review |
| Singer AD et al. | 2016 | Skeletal Radiol. | Review |
| Angelini A et al. | 2023 | J Clin Med | Review |
| Cavalcante MM et al. | 2021 | Foot | Review |
| Murai NO et al. | 2018 | Radiol Clin North Am | Review |
| Rammelt S et al. | 2011 | Oper Orthop Traumatol | Review |
| Campanacci DA et al. | 2021 | Foot Ankle Clin | Review |
| Kennedy JG et al. | 2016 | Foot Ankle Spec | Review |
| Ring A et al. | 2016 | Front Surg | Review |
| Mascard E et al. | 2017 | EFORT Open Rev | Review |
| Jeon JY et al. | 2016 | Clin Imaging | Review |
| Liu XW | 2015 | Int J Clin Exp Med. | Review |
| Rhee JH et al. | 2008 | Magn Reson Imaging Clin North Am | Review |
| Kilgore WB et al. | 2005 | Foot Ankle Clin | Review |
| Papagelopoulos PJ et al. | 2004 | Foot Ankle Clin | Review |
| Grieser T | 2018 | Radiologe | Review |
| Schatz J et al. | 2010 | Top Magn Reson Imaging | Review |
| Haas Y et al. | 2022 | Radiologie (Heidelb) | Review |
| Toepfer A et al. | 2012 | Orthopade | Review |
| Toepfer A et al. | 2024 | Foot Ankle Orthop | Review |
| Grieser T et al. | 2018 | Radiologe | Review |
| Kiss J et al. | 2014 | Magy Onkol | Review |
| Caro-Domínguez P et al. | 2017 | Pediatr Radiol | Case series too small |
| Chou LB et al. | 2009 | Foot Ankle Int | Case series too small |
| Buchner M et al. | 2005 | Chirurg | Case series too small |
| He Z et al. | 2016 | Chin J Med Imaging Technol | Chinese article |
| Wang C et al. | 2014 | Tumor | Chinese article |
| Jiang ZM et al. | 2003 | Zhonghua Bing Li Xue Za Zhi | Chinese article |
| Delgado Cedillo EA et al. | 2007 | Acta Ortop Mex | Spanish article |
| Toepfer A et al. | 2018 | BMC Cancer | Duplication |
| Froeb D et al. | 2012 | Klin Padiatr | Duplication |
| Pollandt K et al. | 2003 | Z Orthop Ihre Grenzgeb | Duplication |
| Matsuoka M et al. | 2024 | Foot Ankle Surg | Overlapping database, longest time interval included |
| Wang Z et al. | 2013 | Cancer Manag Res | Overlapping database, longest time interval included |
| Kask G et al. | 2021 | Bone Joint J | Overlapping database, longest time interval included |
| Shaylor PJ et al. | 2000 | Foot | Overlapping database, longest time interval included |
| Metcalfe JE et al. | 2004 | Foot Ankle Surg | Overlapping database, longest time interval included |
| Yang P et al. | 2017 | Ann R Coll Surg Engl | Overlapping database, longest time interval included |
| Azzopardi C et al. | 2021 | Br J Radiol | Overlapping database, longest time interval included |
| Laitinen MK et al. | 2021 | Bone Joint J | Overlapping database, longest time interval included |
| Weger C et al. | 2013 | Int Orthop | Overlapping database, longest time interval included |
| Furtado S et al. | 2015 | Bone Joint J | Overlapping database, longest time interval included |
| Zeytoonjian T et al. | 2004 | Foot Ankle Int | Overlapping database, longest time interval included |
| Cesari M et al. | 2019 | Pediatr Blood Cancer | Overlapping database, largest patient cohort included |
| Anninga JK et al. | 2013 | Virchows Arch | Overlapping database, largest patient cohort included |
| Casadei R et al. | 2004 | Clin Ortop Relat Res | Overlapping database, largest patient cohort included |
| Özger H et al. | 2018 | J Surg Oncol | Case series too small due to defining ACTs as low-grade chondrosarcoma |
| Özer D et al. | 2017 | J Foot Ankle Surg | Case series too small due to defining ACTs as low-grade chondrosarcoma |
